# A webinar series to educate applicants about the UK academic foundation programme: a longitudinal cross-sectional study

**DOI:** 10.1186/s12909-022-03961-z

**Published:** 2022-12-29

**Authors:** Luke F Western, Lawrence A Gillam, Connor JS Moore, Kitty HF Wong, Robert Hinchliffe

**Affiliations:** 1 Oxford University Clinical Academic Graduate School, Oxford, England; 2 University of Leeds, Leeds, England; 3 Bristol Centre for Surgical Research, University of Bristol, Bristol, England

**Keywords:** Training support, Distance education, Mentoring, Medical education

## Abstract

**Background:**

The UK academic foundation programme (AFP) is a competitive programme for medical graduates and forms the initial stage of the integrated clinical academic pathway. The application is complex and targeted education is beneficial. As online technologies improve, virtual medical education is becoming more common. Currently, webinar education, particularly that of webinar series, are poorly evidenced. An online course was created to investigate the acceptability and effectiveness of webinars for medical education.

**Methods:**

A six-part, one-hour sessional webinar course was developed following a focus group with academic foundation doctors. A pre- and post-course cross-sectional questionnaire study evaluated participant demographics, webinar opinion and self-rated understanding of the AFP via Google Form (Google, USA). Where applicable a five-point Likert scale (1-Strongly disagree to 5-strongly agree) was utilised and analysis using non-parametric paired statistical analysis.

**Results:**

Medical students (*n*=303) from 35 UK universities completed the pre-course questionnaire. Most students had not received targeted education on the AFP. They rated webinars useful for education (mean=4.2 s.d. 0.7).

After the course, participants (*n*=66) expressed it was significantly convenient (mean=4.7), effective (mean=4.7) and suitably interactive (mean=4.4) (*p*<0.001 compared to neutral). Participants preferred short sessions over multiple days to the concept of a full-day event (mean=4.6 vs 3.1, *p*<0.001).

Paired analysis of participants completing both forms (*n*=47) demonstrates a significant increase in self-rated understanding of AFP content, portfolio building, application process, acute clinical scenarios, interview technique and overall confidence in acquiring an AFP post (*p*<0.001).

Follow-up identified 43 participants who completed the course were successful in their AFP application. This represents 7.8% of all successful AFP applicants in 2021.

**Conclusions:**

This study evidences an accessible and effective webinar series for AFP education. Comprehensive webinar courses for similar topics and demographics may provide valuable utility in the provision of future medical education.

**Trial registration:**

Ethics requirements were waived for this study by Bristol University Ethics Committee. All participants in this study consented for anonymous use of their data. As such the trial is not registered.

## Background

The UK academic foundation programme (AFP) forms the initial stage of the integrated clinical academic pathway for postgraduate physician doctors. These posts can also include opportunities in education and leadership and management, thus from 2021, are collectively referred to as Specialist Foundation Programmes (SFP). These posts provide 5% of foundation trainees with protected academic time, additional related education and affiliation to academic networks within their deanery [1. ]. These positions are increasingly competitive, with 22% of trainees applying for an AFP post in 2020 [2. ]. Unlike the traditional foundation program (FP), an extensive application form and face-to-face interview is required. Understanding the various steps and key attributes are major factors in succeeding [1. ].

Many universities attempt to teach their students about the AFP and its application. Unfortunately, the success of this is variable and can be influenced by a number of institution specific factors [3. , 4. ]. In response to this, various courses have been developed [5. ]. Historically these have been held face-to-face, been in central locations and often charged fees (usually £50-150). This generates inequality in accessibility.

Online mediums for medical education have become increasingly common in recent years [6. ]. Webinars have evolved as a popular medium, largely due to reduced logistical issues, associated costs, and being more widely accessible [7. –9. ]. SARS-COV2 has presented a number of challenges, namely, delivering safe education due to necessity of social distancing. Online education evades these issues and has been widely utilised throughout the pandemic. Despite having become a major tool for medical education among medical schools and postgraduate organisations, there is sparce evidence in the literature discussing their educational utility [10. ]. Indeed, previous medical application education studies have previously focused on a single session with no further follow-up [5. , 8. , 9. , 11. ].

The aim of this study is to explore the utility of a webinar series for medical education and gain longitudinal feedback. A free to access AFP educational webinar course was created.

## Methods

Six AFP physician doctors completed a focus group discussion, where a webinar series was established as the preferred design based on previous educational experiences. The course focused on building understanding of AFP application process, how to build a portfolio and how to approach each stage of the interview (personal station, acute clinical scenario management, and the academic station). The “Access the AFP” course took place over a three-month period from August to November 2020, and consisted of six, one hour teaching sessions. There was also a single one-to-one online mock interview with personalised feedback available to participants. The sessions and interviews were hosted and taught by AFP doctors live via Zoom (Zoom Technologies, China), allowing for interactive participation. Cameras and microphones of presenters only were active to optimise bandwidth. Participants could ask questions by turning on microphones or typing into the chatroom.

Any medical student was eligible to attend the course, however it was recommended particularly to penultimate and final year students. The course was advertised through social media platforms Facebook (Meta Platforms Inc, USA) and Twitter (Twitter Inc, USA) to student pages. Universities were also asked to circulate fliers via email to their students.

The study used a descriptive longitudinal cross-sectional questionnaire to evaluate participants over the course of the webinar series. Data was collected from participants via Google Form (Google, USA) questionnaires. All participants who attended the first and last two sessions were eligible and invited to complete the pre-course and post-course questionnaire respectively. A later follow-up questionnaire was circulated in February 2021 to all students who signed up to the final two sessions to investigate AFP allocation among course participants. Participants included in the study consented in each form for anonymised data to be analysed and shared. Data included participant demographics, opinions of webinar education and AFP understanding. The focus of the questionnaires was to establish participants self-rated understanding of the AFP and opinions on webinar-based education more broadly. Where applicable a five-point Likert scale (1-Strongly disagree to 5-strongly agree) was utilised. All fields on the form were mandatory resulting in no missing data in the dataset.

In statistical analysis, normality of data was assessed using the Shapiro-Wilk test. Analysis of the data utilised an unpaired statistical test or a paired approach where appropriate. In questions where a comparable question was not present in both questionnaires, results were analysed compared to neutral (3, on the Likert scale). Statistical significance was set at *P*>0.05. The software IBM SPSS Statistics 26 was used for statistical analysis and Microsoft Excel used to present data.

## Results

Three-hundred and three students participated in the pre-course questionnaire and consented for inclusion in this study. Sixty-six participants completed the post-course questionnaire, with 47 participants having completed both surveys.

The course received participants from 35 different medical schools in the UK (60.7% female, 36.7% male, 2.4% did not specify). Most participants were in their final year of medical school (46.7%), 29.5% were in their penultimate year, 13.1% in pre-clinical years and 10.7% were in an intercalated year. Participants had a variety of higher education backgrounds, including no previous degree qualification (39%) BSc (36.9%), BA (10.4%), MSc (8.3%), MRes (3.9%) and PhD (1.5%). The majority of participants had used a webinar platform prior to this course (86.6%).

The data was found to be non-normally distributed (Shapiro-Wilk *P*<0.001) as well a significantly skewed distribution in multiple questions. Therefore, data was analysed using non-parametric tests. Participants general acceptability of webinars at pre-course questionnaire was generally positive (Table 1).

**Table 1:** Pre-course attitudes to webinar education. Summary of participant responses to questions isolated to the pre-course questionnaire using a 5-point scale (1=Strongly Disagree, 2=Disagree, 3=Neutral, 4=Agree, 5=Strongly Agree). An unpaired single sample t-test is used to compare answers to a neutral stance

**To what extend do you agree with the following statement:**	**Mean ± SD**	**Mean Difference**	***P*-Value**
Webinars are useful platforms for learning medical topics	4.22 ± 0.69	1.22	<0.001
Webinars are more convenient than face-to-face courses	3.83 ± 1.01	0.83	<0.001
I would be more likely to attend a webinar than a face-to-face teaching course	3.62 ± 1.10	0.62	<0.001
I would not have accessed AFP teaching material if there was a cost	4.10 ± 0.92	1.10	<0.001

Prior to the course, most participants had only gained AFP knowledge from online websites (48.8%). Other sources of information were friends (13.1%), university student societies (6.5%), university talks (6.3%) or books/articles (7.5%). Only 1.2% of participants had attended another AFP course and 16.7% of participants had received no previous source of AFP information. Participants responded negatively regarding whether their medical school had provided useful AFP information (2.57 ± 0.96). By the end of the course (3 months) a higher proportion of participants had interacted with university talks (13.3%) and other courses (8.2%). Only 11.2% said they used no other resources for AFP preparation than the Access the AFP webinar course.

Questions applicable to pre- and post-course questionnaire focusing on self-reported confidence, understanding and application preparedness were all significantly improved (*p*=<0.05) after the course with both paired and unpaired statistical analysis (Table 2).

**Table 2:** Analysis of webinar educational outcomes. Summary of participant responses to statement using a 5-point scale (1=Strongly Disagree, 2=Disagree, 3=Neutral, 4=Agree, 5=Strongly Agree). Both pre-course and post course mean and standard deviations are provided with associated unpaired and paired statistical analysis

**To what extend do you agree with the following statement:**	**Pre-Course (*n*=330)**	**Post-Course Paired Analysis (*n*=47)**		**Post-Course Unpaired Analysis (*n*=66)**	
	**Mean±SD**	**Mean±SD**	***P*-Value**	**Mean±SD**	***P*-Value**
I feel confident I would get an interview for an AFP	2.93 ± 0.93	3.94 ± 0.73	<0.001	3.96 ± 0.74	<0.001
I feel confident I would get a place on an AFP	2.59 ± 0.79	3.45 ± 0.78	<0.001	3.43 ± 0.78	<0.001
I understand the AFP application process	3.02 ± 1.06	4.47 ± 0.69	<0.001	4.46 ± 0.66	<0.001
I understand the UK academic clinical pathway	3.10 ± 0.98	4.28 ± 0.80	<0.001	4.24 ± 0.76	<0.001
I am aware of the differences between academic units’ programmes such that I can tailor my application choice to my interests	2.71 ± 1.07	4.06 ± 0.90	<0.001	3.34 ± 0.82	<0.001
My university provides useful information into the UK clinical academic pathway	2.57 ± 0.96	3.21 ± 1.27	0.003	3.63 ± 1.23	<0.001
I have someone who is completing an AFP who I could discuss the application process with	2.73 ± 1.30	3.57 ± 1.28	0.001	3.99 ± 1.25	<0.001
I understand how to prepare a strong CV and portfolio	2.69 ± 1.02	3.98 ± 0.90	<0.001	4.06 ± 0.87	<0.001
I feel confident in how to construct white space questions	2.25 ± 0.97	4.04 ± 0.88	<0.001	3.85 ± 0.86	<0.001
I am confident in critically appraising academic work	2.67 ± 1.06	3.81 ± 0.90	<0.001	3.97 ± 0.89	<0.001
I feel confident discussing the management of acute clinical situations	2.78 ± 1.03	3.92 ± 0.78	<0.001	3.96 ± 0.75	<0.001
I understand ethical principles that apply to clinical and academic situations	3.03 ± 1.04	3.94 ± 0.90	<0.001	4.25 ± 0.84	<0.001
I understand the structure of the AFP interviews	2.48 ± 1.01	4.26 ± 0.74	<0.001	4.22 ± 0.70	<0.001
I have a plan of how to prepare for the AFP application process	2.53 ± 1.02	4.19 ± 0.77	<0.001	3.96 ± 0.73	<0.001
I feel I could perform well in an AFP interview	2.71 ± 0.92	3.957 ± 0.72	<0.001	4.42 ± 0.73	<0.001

Participants reported significantly positive feedback on course convenience, effectiveness and interaction (*p*=<0.01 when compared to neutral score of 3) and indicate preference for a staggered course in comparison to a whole day course (Table 3).

**Table 3:** Participant feedback of webinar. Summary of participant responses to statements isolated to the post-course questionnaire using a 5-point scale (1=Strongly Disagree, 2=Disagree, 3=Neutral, 4=Agree, 5=Strongly Agree). An unpaired single sample t-test is used to compare answers to a neutral stance

**To what extend do you agree with the following statement:**	**Mean ± SD (*n*=66)**	***P*-Value**
I found this webinar series convenient	4.75 ± 0.56	<0.001
I would be more likely to attend a webinar than a face-to-face teaching course	4.50 ± 0.78	<0.001
This webinar series was an effective method to learn about the AFP	4.71 ± 0.58	<0.001
This webinar series has increased my understanding of the AFP	4.77 ± 0.43	<0.001
This webinar series has increased my interest in applying to the AFP	4.60 ± 0.65	<0.001
I found it easy to interact with presenters	4.40 ± 0.80	<0.001
I found it easy to have my questions answered	4.44 ± 0.47	<0.001
I would recommend this webinar series to others	4.75 ± 0.47	<0.001
I prefer having multiple webinars to cover the AFP topic	4.63 ± 0.83	<0.001
I would prefer such a course as a one-day event	3.18 ± 1.50	0.229
I believe that the Access the AFP webinar series has prepared me to apply to the academic foundation program application	4.42 ± 0.63	<0.001

Forty-eight participants responded to a follow-up questionnaire following the release of AFP job offers. Of these, 43 participants were successful in gaining an AFP job. This represents 7.8% of all UK AFP jobs. The majority (77.1%) agreed or strongly agreed that this course was essential preparation for their application. However, the number of course attendees that applied is unknown.

## Discussion

As educational technology improves, online mediums for medical education are becoming more prevalent, such as pre-recorded lectures and live online sessions [12. ]. The SARS-COV2 pandemic escalated the utilisation of online platforms, with many universities abandoning their usual face-to-face teaching. The success of online teaching for medical school education appears inconclusive, as students felt relatively unprepared for their role as doctors in a recent study by Dost et al [13. ]. Conversely, webinars teaching specific information on clinical application processes appears to be effective. These are typically well planned, with education material tailored for online teaching [8. , 9. , 11. ]. At this stage, there appears to be moderate evidence online education can be as effective as face-to-face education, but more objective evidence will be required to conclude this, as many studies, including this one, have used self-reported outcome measures [6. , 10. , 12. ]. In China, there is already an established shift from traditional courses, to massive online open courses (MOOCs) [14. ]. These courses appear to be widely accepted and demonstrate equivocal examination pass rates, and might be an indication of the future direction of medical education globally [14. , 15. ]. This study demonstrated a significant improvement in self-reported understanding for each domain related to the academic foundation programme and at least a 7.8% representation of all successful UK AFP candidates completed this course. This supports the implied notion that a planned and targeted online webinar series is effective at teaching medical students. However, further research into online education for other topics and subsequent medical student preparedness would be required to generalise webinar education.

In addition to effectiveness, online education should be considered acceptable by students to maximise engagement. A recent systematic review by O’Doherty et al explains the importance of attitude towards the online education platform in ensuring engagement and learning outcomes [12. ]. There has been a gradual increase in acceptability of webinar platforms in similar such studies over recent years. In 2019, students preference of webinar learning over face-to-face learning was 32% [8. ]. In 2020, preference is demonstrated at 52.2% and the results of this study describe a preference of 58.7% [9. ]. As online educational tools are integrated into the culture of educators and students, it is likely that acceptability, engagement and utility of online education will increase [6. , 12. , 15. ].

In comparison to similar literature, this course utilised a unique modular structure over multiple sessions to increase accessibility [8. –10. ]. Participants received this well, with a significant result indicating preference to multiple sessions (4.63±0.83). A modular structure gives more opportunities for attendance, doesn’t require a full day of commitment, and provides the participant the opportunity to attend only the sessions they feel are beneficial to them. Additionally, shorter, spaced learning may provide better learning outcomes as students have more rest time to synthesise information [16. ].

This course demonstrates high accessibility, with representation from 35 UK medical schools with 95% of participants finding the course convenient in the post-course questionnaire. Face-to-face education has inherent limitations in regard to accessibility. Medical students may not be available to travel to central locations due to university commitments or may be situated abroad. Furthermore, face-to-face education typically ensues additional costs due to required physical space, printouts, catering, and cleaning. These can be avoided through online methods [17. ].

Accessibility of medical education is important, as the lack of exposure to mentors decreases focused career planning and the likelihood of specialty or academic career commitment [3. , 4. , 18. , 19. ]. The integrated clinical academic pathway was created to increase the number and quality of clinicians entering clinical academic careers [1. ]. Many participants felt poorly prepared and received little information from their universities. This lack of in-house mentorship could reflect the discrepancy of AFP applications between universities. For example, in 2020 the average AFP application rate among medical students was 20.9%, but medical schools known to discuss AFP applications more significantly such as Oxford, Cambridge and Imperial received application rates of 41%, 40.9% and 42.5% respectively [2. , 4. ]. More accessible and equivalent access to AFP education could further promote the clinical academic career and encourage promising young clinicians who previously may not have engaged with academia due to lack of mentoring and exposure.

Strong communication and engagement are important in achieving better outcomes and collaborative learning [20. ]. This study demonstrated a significant proportion of participants found it easy to interact with the presenters. This was achieved via a live chat room with a dedicated faculty member answering, frequent opening to live questions, promotion of student discussions during content teaching and being available via email for follow-up questions. Dost et al specifically mentioned medical students found university education was best received where group discussion and chat room facilities were available live, as this increased engagement and satisfaction with the education [13. ]. However, some people do prefer face-to-face interactions and a recent paper by Hameed et al discusses the future development of hybrid courses and clinical meetings, with some participants present and others virtually attending. This may provide solutions to different peoples learning needs and preferences, while maintaining reasonable cost-effectiveness [17. ].

### Limitations

As with many questionnaire-based studies, loss to follow-up was significant in this study. The questionnaire was emailed directly to participants after the course. No incentives were utilised to encourage participation which may have reduced engagement. Non the less, there is a large sample size when compared to similar literature, and the smaller post-course sample size likely reflects a proportion of participants who did not complete the course, as well as loss to follow-up [7. –9. ].

There are proportionally more females in the study. It has been previously cited that females tend to provide more positive feedback regarding webinar education than male colleagues, thus potentially influencing outcomes [21. ]. The methodology of this study is not optimised to establish if the gender distribution is due to higher likelihood of female participation in webinar education or simply reflecting the demographics of medical students at UK universities (approximately 59% female) [22. ].

It should be noted that there is an inherent selection bias when evaluating the utility of webinar teaching by utilising a webinar course. Naturally, participants may have a more positive bias as they have actively sort out the platform. However, this may be partially mitigated by SARS-COV2 pandemic, as those who may have wanted to attend face-to-face education on such a topic were largely unable to.

This study also utilises somewhat subjective, self-reported understanding. Such data may be prone to demand characteristics. However, this is in keeping with similar webinar research [7. –9. ]. Furthermore, the post job allocation questionnaire was clearly prone to response bias, as those with offers were by far the majority of responders. This is why it was not possible to establish application to offer rate for this webinar course. Future research should explore the specific AFP application outcomes of participants who partake in a webinar course vs a face-to-face course. This may go some way to demonstrating more objectively if webinars are a more effective alternative for face-to-face medical education.

## Conclusion

This study demonstrates a webinar course is an effective, acceptable, and accessible method of delivering medical education related to the AFP. As webinars and online teaching methods in medical education become more common place, further research should be conducted to establish the relative effectiveness in comparison to face-to-face modalities.

## Data Availability

The datasets generated and/or analysed during the current study are not publicly available due to an effort to maintain individual confidentiality but are available from the corresponding author on reasonable request.

## Abbreviations

AFP: United Kingdom academic foundation programme
